# Computel: Computation of Mean Telomere Length from Whole-Genome Next-Generation Sequencing Data

**DOI:** 10.1371/journal.pone.0125201

**Published:** 2015-04-29

**Authors:** Lilit Nersisyan, Arsen Arakelyan

**Affiliations:** Group of Bioinformatics of the Institute of Molecular Biology of the National Academy of Sciences of the Republic of Armenia, Yerevan, Armenia; Queen's University Belfast, UNITED KINGDOM

## Abstract

Telomeres are the ends of eukaryotic chromosomes, consisting of consecutive short repeats that protect chromosome ends from degradation. Telomeres shorten with each cell division, leading to replicative cell senescence. Deregulation of telomere length homeostasis is associated with the development of various age-related diseases and cancers. A number of experimental techniques exist for telomere length measurement; however, until recently, the absence of tools for extracting telomere lengths from high-throughput sequencing data has significantly obscured the association of telomere length with molecular processes in normal and diseased conditions. We have developed Computel, a program in R for computing mean telomere length from whole-genome next-generation sequencing data. Computel is open source, and is freely available at *https://github.com/lilit-nersisyan/computel.* It utilizes a short-read alignment-based approach and integrates various popular tools for sequencing data analysis. We validated it with synthetic and experimental data, and compared its performance with the previously available software. The results have shown that Computel outperforms existing software in accuracy, independence of results from sequencing conditions, stability against inherent sequencing errors, and better ability to distinguish pure telomeric sequences from interstitial telomeric repeats. By providing a highly reliable methodology for determining telomere lengths from whole-genome sequencing data, Computel should help to elucidate the role of telomeres in cellular health and disease.

## Introduction

Telomeres are nucleoprotein structures, located at the ends of eukaryotic chromosomes, aimed at protecting chromosome ends from degradation and helping them to overcome the so called "end replication problem" [1–3]. Telomeric DNA sequences consist of short tandem repeats, the composition of which differs between different organisms. The range of telomere lengths in the same organism also depends on the tissue type [4]. In most somatic cells, telomere length gradually shortens with cell divisions, eventually leading to replicative cell senescence [5]. This attrition may be compensated by telomere elongation by telomerase, an RNA dependent reverse transcriptase, which is highly expressed in germline cells and the majority of cancer cells [6].

Changes in telomere length dynamics have been shown to be associated with various types of cancers [7, 8]. On the other hand, short telomeres are shown to accompany a number of age-related diseases, such as atherosclerosis, heart diseases, ulcerative colitis, liver cirrhosis, premature ageing syndromes, etc. [9–12]. This association is partially explained by telomere position effect (TPE), the phenomenon of reversible silencing of genes located near telomeres dependent on genes’ distance to telomeres and telomere lengths [13, 14].

In the light of their importance to cell fate regulation, cancers and various age-related diseases, telomeres have been subject to extensive studies. For this purpose, several experimental techniques have been developed for measuring telomere length in cells, such as terminal restriction fragment analysis (TRF), quantitative PCR (qPCR), quantitative fluorescent in situ hybridization (qFISH), etc [15]. With the exception of qFISH, these methods do not report the telomere length at individual chromosome ends, but rather its mean or absolute value per single genome. Even though individual telomere lengths are important characteristic for genomic stability analysis, the mean telomere length has been successfully used in a large number of studies as a surrogate marker for telomere attrition state and its association with diseases [16–19]. However, these methods are time- and resource- consuming, and have a number of limitations and drawbacks [15].

With the advent of next-generation sequencing (NGS) approaches it has become possible to obtain whole-genome sequences of individual organisms [20]. Consequently, attempts have been made to develop approaches for telomere length measurement techniques based on these data. Recently, two methods were proposed for this, both of them based on count of short-reads containing telomeric repeats [21, 22]. From those, only TelSeq is released as software available for free download and use [22]. While these approaches were shown to be appropriate for analysis of mean telomere length in genome [23], they also have possible drawbacks, such as dependence on repeat count threshold, which can bias telomere length estimates, and NGS sequencing errors, which can distort the telomeric lookup pattern.

We have developed Computel—an alternative, alignment-based approach for telomere length estimation using whole-genome NGS data. Herein, we have performed its extensive validation using both synthetic and experimental data. Comparison of Computel with TelSeq showed that our approach outperforms the latter and allows for more flexibility and convenience of telomere length estimation in different genomes.

## Materials and Methods

### Algorithm description

#### General workflow

Computel is written in R 3.0.3 and performs command line calls to the following programs during execution: Bowtie 2–2.1.0, Samtools 0.1.19, and Picard tools 1.108. Computel can be called both from R environment and through command line, with an Rscript front-end available for Windows and UNIX type systems. Detailed information about Computel installation and usage is available in its manual (see S1 Information).

**Fig 1:**
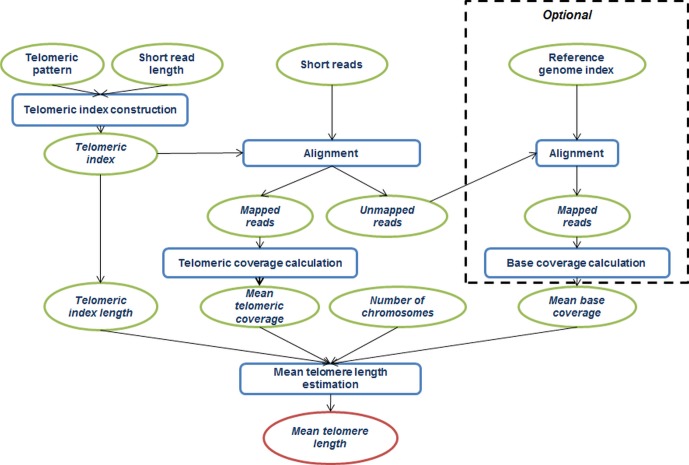
Schematic representation of the Computel algorithm for mean telomere length estimation. Computel takes whole-genome NGS short-reads as input; maps them to the telomeric index built based on user-defined telomeric repeat pattern and the read length; and calculates the mean telomere length based on the ratio of telomeric and reference genome coverage, the number of chromosomes, and the read length.

The general workflow of mean telomere length estimation by Computel is schematically represented in Fig 1. It consists of the following steps: (1) building a telomeric index, (2) mapping reads to the telomeric index, (3) coverage calculation at the telomeric index, (4) determination of mean coverage at reference genome (optional), (5) estimation of mean telomere length. Each of these steps is described in detail in the following subsections.

#### Building a telomeric index

**Fig 2:**
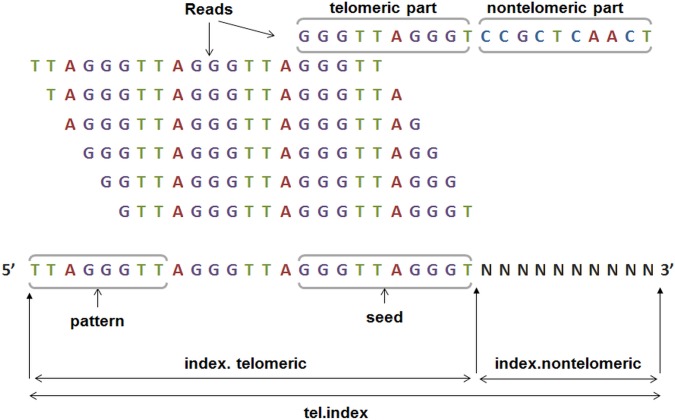
An example of sequence structure of telomeric index for telomeric read alignment. Telomeric pattern is “TTAGGG” (human); read length = 20 nt; seed length (*min*.*seed* option) = 10 nt. The top read contains a non-telomeric region, which will be aligned to the non-telomeric tail of the index, the rest of the reads are six possible cyclic permutations of pure telomeric repeats.

The telomeric index is built using the *bowtie2-build* program. The index is designed such a way that any read consisting of telomeric repeat patterns can map uniquely to the index. It is also important to take into consideration reads that contain telomeric repeats only partially: theoretically, we would like to also capture the reads originating from chromosome regions located at the junction of telomeric and immediate subtelomeric sequences. For this reason, the telomeric index has an additional 3'-end tail containing ambiguous nucleotide (N) bases to which any sequence can map (Fig 2). Note that the N-tail is attached only to the 3' end of the index, to minimize the number of captured reads containing interstitial telomeric repeats [24].

The pseudocode for generation of the telomeric index is presented below:

Let *pattern* be the sequence of telomeric repeat pattern bases;

Let *pl* be the length of the *pattern;*

Let *rl* be the read length;

Let *min*.*seed* be the minimum number of telomeric read bases in the mapped reads;

Let *tel*.*index* be the sequence of the telomeric index;

Let *index*.*telomeric* be the region of the index containing telomeric repeats;

Let *index*.*nontelomeric* be the region of the index containing ambiguous bases {N};

The sequence of *tel*.*index* is computed as follows:

length(*index*.*telomeric*) = *rl* + *pl*—1

*count*.*pattern* = int(length(*index*.*telomeric*)/*pl*)

*count*.*substring* = length(*index*.*telomeric*)/*pl* % *pl*

*index*.*telomeric* = concatenate(*count*.*pattern* * *pattern*,

*pattern*[1:count.substring])

*index*.*nontelomeric* = (*rl*—*min*.*seed*) * {N}

*tel*.*index* = concatenate(*index*.*telomeric*, *index*.*nontelomeric*)

*tel*.*index* sequence is supplied to the *bowtie2-build* program to build the index files. An example of *tel*.*index* sequence is given in Fig 2.

#### Aligning short-reads to the telomeric index

Paired- or single-end short-reads are aligned to the telomeric index with the program bowtie2-align. By default, the alignment is performed with the Bowtie 2 preset options for—*end-to-end* alignment, with—*very-sensitive* mode,-*N* set to 1 (to allow mismatches in a seed alignment), and-*L* ranging between 6 and 22, which is calculated automatically, depending on read length. The choice of these options is explained in the S2 Information, section *The Influence of the Bowtie 2 alignment parameters on telomere length estimation*.

The resulting alignment is stored in a SAM file.

#### Reference genome (base) coverage calculation

For reference genome coverage calculation, the generated SAM file is split into two SAM files containing mapped and unmapped reads. The SAM file containing unmapped reads is converted back to a FASTQ file using the Picard *SamToFastq* tool. Unmapped reads are then aligned to the reference genome with Bowtie 2 default options, sorted and used for base coverage (*base*.*cov*) calculation with the Samtools *depth* command.

Mapping short-reads to reference genome can be time-consuming. However, without significant loss in accuracy (data not shown), the base coverage can be estimated as:

base.cov=(total number of reads)*(rl)/(total genome length),

and supplied to Computel as an argument.

#### Telomeric coverage calculation and mean telomere length estimation

The SAM file for mapped reads is sorted and the distribution of coverage per base for the telomeric index is calculated using the Samtools *depth* command.

We have used the mean value of coverage at each base as a point estimate for coverage at telomeric index (*tel*.*cov*). The relative coverage at telomeric index compared to the reference genome is computed as *rel*.*cov = tel*.*cov* / *base*.*cov*. Finally, the mean telomere length (MTL) is estimated as:

MTL=(mean(rel.cov))*(rl+pl−1)/(2*n_chr),

where the number 2 in the denominator accounts for the two chromosome ends, and *n_chr* is the number of chromosomes in the haploid genome. The rest of the variables are explained above.

### Algorithm validation with synthetic data

In order to estimate the algorithm performance, we carried out a series of telomere length calculations using synthetic data. For this purpose, we have taken a ~200 kb fragment of human reference chromosome 1 (GRCh37) from the NCBI Genome database. This fragment did not contain either pure or interstitial telomeric repeats [24], nor did it contain ambiguous bases; it was used for two purposes. First, it served as a reference genome for base coverage estimation (see below). Second, telomeric sequences consisting of human telomeric TTAGGG or CCCTAA repeats with known lengths were attached *in silico* to both ends of this chromosome fragment. The telomeric sequence lengths were randomly chosen from a normal distribution with mean 10 kb and standard deviation 7 kb. The resulting sequences were used to generate artificial short-reads using the ART tool for Illumina sequencers [25].

The following testing scenarios have been exploited:

- short-reads of different lengths from the set {20, 36, 51, 76, 100, 150 nt};
- short-reads with different insert sizes from the set {200, 300, 500 nt};
- short-reads with different coverage values from the set {0.1, 0.5, 1, 2.5, 5, 10, 30};
- paired-end and single-end short-reads.

The 200 nt insert size was not considered for 100 nt or 150 nt length reads; and the 300 nt insert size was not considered for 150 nt length reads.

### Performance comparison with TelSeq

We compared the performance of Computel to that of TelSeq [22]. Both software were used with their default settings, unless otherwise stated.

TelSeq computes telomeric length with the formula *l = t*_*k*_ *sc*, where *l* is mean telomere length, *t*_*k*_ is the abundance of telomeric reads, *s* is the fraction of all reads with GC composition between 48% and 52%, and *c* is a constant for the genome length divided by the number of telomere ends [22]. First, we performed comparisons based on the settings for short-reads generation taken from the original TelSeq paper [22]. Briefly, human chromosome 1 of the GRCh37 genome assembly was used as a reference. Terminal sequences of 30 kb length, including N-bases and telomeric repeats, were removed from each end of the chromosome and replaced with the same length of telomeric repeats. Illumina short-reads were generated with the SimSeq tool (*https://github.com/jstjohn/SimSeq*) using the following parameters: *-1 100–2 100—insert_size 500—insert_stdev 200*, with coverage equal to 0.4x (498,501 reads), 2x (2,492,506 reads), or 10x (12,462,531 reads), and with duplication rate fixed at 5% for all coverages. Each setting was repeated 5 times. Mean telomere length was measured with TelSeq using exactly the same settings described in the original paper [22]. Because the genome length constant for telomeric GC content is hard-coded in the TelSeq software, we computed this parameter for chromosome 1 (21,722,000 for chromosome 1 instead of 332,720,800 for the total genome) and recompiled TelSeq with the new value (see S3 Information). The mean telomere length estimates by TelSeq were compared to Computel’s estimates.

To account for read length variation, we have repeated the settings described above, with fold coverage equal to 2x, and for read lengths equal to 36 nt (6,923,628 reads), 76 nt (3,279,613 reads), 100 nt (2,492,506 reads), and 150 nt (1,661,671 reads). TelSeq *k* threshold was kept at default value of 7 for all read lengths, except for 36 nt, for which we have tested *k* values equal to 4, 5, and 6.

Finally, we have assessed the performance of Computel and TelSeq depending on short-read generation algorithms. For this, we have generated 100 nt length short-reads with 0.4x (498,501 reads), 2x (2,492,506 reads), and 10x (12,462,531 reads) coverage using another short-read generation tool, ART Illumina read generator, with its default parameters [25].

### Measures of telomere length estimation accuracy and statistical analysis

The accuracy of telomere length estimation was evaluated with the following criteria:

- Mean of relative error (MRE), and standard error (SE), where relative error of estimated (EL) over actual telomere length (L), is the ratio (EL-L)/L;
- Root mean squared error (RMSE), which represents the variance of mean telomere length estimation error;
- Coefficient of determination, R^2^, which is the estimate of quality of linear fit (goodness of fit) between estimated and actual mean telomere lengths.

Paired t-tests were used for pairwise comparisons, single factor analyses were performed using ANOVA, and, finally, the effects of several factors on accuracy of estimation were assessed using multi-factor ANOVA. P values less than 0.05 were considered significant. Statistical calculations were performed using SPSS 19.0 (IBM Inc, USA).

### Validation with experimental data

In order to validate Computel performance with experimental data, we downloaded whole-genome sequencing data for paired tumor and healthy tissues of 5 neuroblastoma and 2 osteosarcoma patients [21], from EGAD00001000135 and EGAD00001000159 datasets deposited at the European Genome-phenome Archive (*http:www.ebi.ac.uk/ega*). For these samples, differences in telomere length between normal and tumor tissues have previously been estimated using quantitative real time PCR.

We have computed telomere lengths for these datasets with Computel and TelSeq and validated the results against experimentally obtained data, described by Parker et al [21].

## Results

### Algorithm performance

We have assessed the accuracy of mean telomere length estimation by Computel in a series of computations performed on "synthetic chromosomes" with telomeres of known length attached to their ends. The lengths of attached telomeres were randomly chosen from a normal distribution with mean 10 kb and standard deviation 7 kb. The range (min-max) of generated telomeres were 194.5–21138 bp for single-end reads, and 387.5–24169.5 bp for paired end reads, respectively.

**Fig 3:**
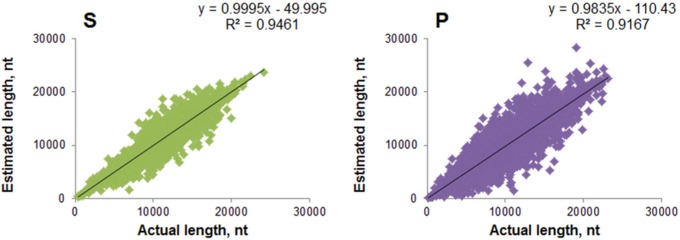
Correlation between actual and estimated mean telomere lengths. S—single-end reads, P—paired-end reads. Estimation of mean telomere length was performed with reads generated from 200 kb length region of human chromosome 1, with telomeres attached to both its ends with lengths sampled from a normal distribution with mean 10 kb and SD 7 kb. The minimum-maximum range of the generated telomere lengths were: 194.5–21138 bp for single-end reads, and 387.5–24169.5 bp for paired-end reads. The read length, insert size and fold coverage ranges are described in the *Materials and Methods*.

The results obtained showed very strong linear correlation between actual and estimated telomere lengths in all the experiments, with the quality of linear fit (R^2^) equal to 0.95 and 0.92 for single and paired-end reads, respectively (Fig 3). The mean relative errors (MRE±SE) between estimated and actual telomere lengths were 4±0.01% for single-end reads and 7±0.01% for paired-end reads.

Next, we compared performance of telomere length estimation based on read length, coverage and insert size (in case of paired-end reads). The most accurate estimates for mean telomere length were obtained with single-end short-reads of length 36 nt with fold coverage equal to 30 (MRE ± SE: -0.19±0.1%). The poorest estimate was detected when telomere length was calculated from paired-end short-reads of length 150 nt, insert size 500 nt and coverage 0.1 (MRE ± SE: -3.95±4.9%). Generally, increasing coverage improves the accuracy of the mean telomere length estimation. In addition we noted a drop in accuracy along with the increase of insert size for paired-end reads. For detailed information about performed analyses the reader may refer to S2 Information, section *Performance assessment*.

### Performance comparison with TelSeq

We have compared the accuracy of telomere length estimation by Computel and TelSeq with short-reads generated from human chromosome 1 with 30 kb telomeric sequences attached (see *Materials and Methods*for details) at 0.2x, 2x and 10x coverage. Computel was used with its default settings, while TelSeq source code was modified to make the computations valid for chromosome 1 (see *Materials and Methods,* section *Performance comparison with TelSeq*).

The results obtained indicate that Computel outperforms TelSeq in all the cases. Moreover, TelSeq fails when short-read length significantly deviates from its default value (Table 1), while the accuracy of Computel is not changed significantly in the read length ranges examined. Finally, to compare Computel and TelSeq performance with an alternative short-read generation algorithm, we also used short-reads generated by the ART Illumina tool. Comparison was performed using the same settings as described above, with 100 nt read lengths and coverage values in the range 0.2x, 2x and 10x. In this case, TelSeq significantly underestimated the actual telomere lengths, in contrast to Computel (Table 1).

**Table 1:** Comparison of performance of Computel and TelSeq in mean telomere length estimation from synthetic data.

Read length	Synthetic short-read generation tool	Computel mean telomere length estimate^a^ mean ± SE, kb	TelSeq mean telomere length estimate^a^ mean ± SE, kb
100 nt ^b^	SimSeq ^e^	29.2 ±0.5	28.8 ± 0.5
36 nt (*k* = 4) ^c^	SimSeq	29.6 ± 0.4	47.2 ± 0.5
36 nt (*k* = 5) ^c^	SimSeq	29.6 ± 0.4	47.1 ± 0.5
36 nt (*k* = 6) ^c^	SimSeq	29.6 ± 0.4	7.8 ± 0.1
76 nt	SimSeq	29.8 ± 0.8	31.8 ± 0.8
150 nt	SimSeq	28.9 ± 0.7	NA ^d^
100 nt	ART Illumina [25]	31.1 ± 0.6	24.6 ± 0.6

^a^ The actual telomere length was 30 kb attached to the Chromosome 1.

^b^ The default TelSeq read length.

^c^ For 36 nt read lengths, estimation of telomere length by TelSeq was performed with *k* (threshold of telomeric repeats in short-reads) equal to 4, 5 or 6; For all other read lengths, the default value of *k* = 7 was used.

^d^ TelSeq fails to output results for 150 nt read length.

^e^ SimSeq is available at *https://github.com/jstjohn/SimSeq*.

### Validation with experimental data

We have computed telomere lengths with whole-genome sequences of tumor (D)—normal tissue (N) pairs from five neuroblastoma patients, using both Computel and TelSeq, with their default settings. These samples have been previously analyzed by qPCR and the log fold changes of telomere lengths in tumor over paired healthy tissues were published [21]. In order to compare those results with the estimates obtained by Computel and TelSeq, we computed absolute values of mean telomere lengths and converted them to log_2_ fold change values. The changes in telomere length predicted by Computel and TelSeq were consistent with length changes observed by qPCR (Table 2).

**Table 2:** Log ratios of telomere length estimates by Computel and TelSeq compared to qPCR for five neuroblastoma (D) and matched normal tissue (N) samples.

Sample	qPCR [log_2_(D/N)]	Computel* [log_2_(D/N)]	TelSeq* [log_2_(D/N)]
SJNBL001	GAIN [2.89]	GAIN [2.45]	GAIN [2.27]
SJNBL002	GAIN [3.92]	GAIN [1.61]	GAIN [1.61]
SJNBL009	LOSS [-1.92]	LOSS [-1.12]	LOSS [-1.16]
SJNBL030	LOSS [-3.81]	LOSS [-0.95]	LOSS [-0.99]
SJNBL031	GAIN [5.35]	GAIN [1.22]	GAIN [1.20]

* the absolute values of the mean telomere lengths for tumor and paired healthy tissue computed by Computel and TelSeq are available in the S2 Information, section *Absolute mean telomere length estimation for neuroblastoma samples*.

**Fig 4:**
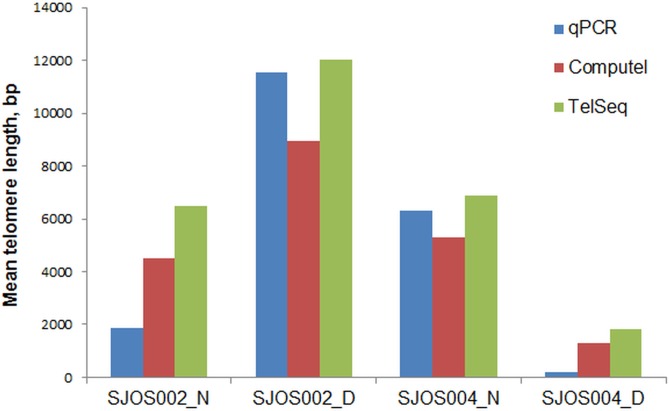
Mean telomere length estimates for osteosarcoma and matched normal tissues by qPCR, Computel and TelSeq. SJOS002_D, SJOS004_D—osteosarcoma tissue samples; SJOS002_N, SJOS004_N—paired healthy tissue samples.

Next we used Computel to estimate telomere lengths for two osteosarcoma samples (SJOS002 and SJOS004) and compared the estimates with absolute qPCR and mTRF values [21]. Computel length estimates were partially consistent with TelSeq estimates and qPCR results, with some differences for each technique (Fig 4). In two out of the four cases, Computel estimates were closer to qPCR values than TelSeq estimates, with TelSeq estimates being closer in the other two cases.

For all the cases of telomere length estimation with experimental data, TelSeq telomere length estimates were by 2–5 kb larger than Computel length estimates. We have hypothesized that this may be the result of TelSeq capturing more reads from interstitial telomeric regions, than Computel. In order to check this, we have retrieved reads from one of the neuroblastoma sample runs (SJNBL001_D-2876158223) that Computel failed to map to the telomeric index, but that contained more than 7 telomeric repeats and were successfully captured by TelSeq. BLAST results showed that some of these reads were similar to available sequences of interstitial regions in human reference genome. The rest of the reads, however were not aligned to any known sequence, but presumably did not originate from telomeric regions, as they do not have canonical telomeric repeat patterns (see S4 Information).

Additionally, we used SimSeq to generate short-reads (5x fold coverage) from subtelomeric 500 kb sequences of human chromosomes [26], available at *http://www.wistar.org/lab/harold-c-riethman-phd/page/subtelomere-assemblies*. From these short-reads, Computel mapped a total of 65 reads to the telomeric index, while TelSeq counted 327 reads. This is consistent with the hypothesis that overestimation of telomere lengths in experimental data by TelSeq compared to Computel can be partially attributed to interstitial telomeric repeats contained in the subtelomeric and other regions of chromosomes.

## Discussion and Conclusions

The importance of telomeres in regulation of cell life and their connection with various age-related diseases and cancers has been known for a long time [9–12]. Their role in regulation of expression of certain genes, as in telomere position effect, has also been investigated in various organisms [13,14]. However, data that can be used to associate telomere length with cell dynamics is scarce, because experimental techniques for telomere length estimation are laborious and expensive, and made it difficult to correlate telomere length with gene expression or epigenetic changes.

Currently, large amounts of high-throughput NGS data for individual organisms are available [27]. Often, they contain not only WGS data, but also data from RNA-Seq, microarrays, or ChIP-Seq, which should make them valuable for associating telomere lengths with gene regulation. It is, however, difficult to calculate telomere lengths from WGS data, because a typical reference genome partially or completely lacks telomeric sequences, with chromosomal termini sometimes being denoted by runs of “N” residues. Moreover, since telomeric regions are very repetitive, traditional methods of alignment of short-reads to genomic sequence are typically confounded in this context by multiple mapping positions of the reads [23]. To overcome these limitations, we have developed the open-source software Computel, which functions by aligning short-reads to a special index, designed in such a way that only telomeric reads map to it in unique positions. Analyses have shown that Computel estimates mean telomere length with high accuracy, and its performance does not significantly depend on read length, short-read type, fold coverage and insert size.

Recently, alternative approaches have been developed for telomere length estimation from WGS data, based on count of short-reads containing a certain number of telomeric repeats [21, 22, 28]. In the cases of [21] and [28], this number was fixed at 4; in case of TelSeq it can vary based on read length [22]. Although TelSeq is a valuable tool, it still has some limitations that we attempted to address with Computel. First, TelSeq sets a threshold for telomeric repeat count, which makes the results of the output dependent on both the threshold and the short-read length; resulting in very poor performance, if read length considerably deviates from 100 nt (e.g., 36 nt or 150 nt). Computel, on the contrary, performs similarly well for all the short-read lengths analyzed (from 20 nt to 150 nt). Secondly, TelSeq performs relatively well on short-read data generated without reading errors in sequences; however, when sequencing errors were introduced with the ART Illumina tool, the accuracy of TelSeq results fell considerably compared to Computel. This is explained by the fact that any single error in a nucleotide sequence distorts the telomeric patterns and affects the count of telomeric reads, while the alignment approach is less sensitive to this type of errors.

An important issue concerned with NGS based telomere length estimation is the fact that there are interstitial telomeric repeats in other regions of chromosomes [24], and it is difficult to distinguish between reads originating from these regions from true telomeric (or immediate subtelomeric) reads. The alignment-based approach utilized in Computel has the ability to reduce the number of such misclassified reads compared to TelSeq, as demonstrated with experimental data (see Results, *Validation with experimental data*). Notably, TelSeq underestimates telomere length in *in silico* experiments, where only “pure” telomeric sequences were present at chromosome ends; whereas with experimental data, where reads from interstitial telomeric sequences are presumably present, TelSeq estimates of telomere length were greater than Computel’s estimates. In addition, when computing telomere lengths, Computel accounts only for the parts of the reads that have been aligned to the telomeric part of the telomeric index (*index*.*telomeric*), thus reducing the bias introduced by subtelomeric repeat-rich regions.

Finally, the hard-coded implementation of several important constants in TelSeq, such as GC-normalized genome length, and the number of chromosomes, makes this software difficult to use for analysis of telomere length of other genomes for researchers with basic programming skills, whereas all parameters of Computel can be easily set in a single configuration file (see S1 Information).

Performance assessment of Computel with experimental data has shown that telomere length estimates correlate with mean telomere length estimated with qPCR and TRF, but deviate to some extent (2–3 kb) in absolute values. In case of TRF, this difference can be attributed to the fact, that TRF also captures subtelomeric regions of chromosomes, thus overestimating telomere length by 2.5–4 kb [15]. On the other hand, estimates of absolute telomere length by qPCR, are very prone to preliminary calibration steps, therefore results obtained in different experimental settings should be compared qualitatively, rather than quantitatively [15]. It is important to note, that existing experimental methods for mean telomere length assessment all have their drawbacks and limitations [29], and, thus, cannot serve as validation methods for computational approaches, such as Computel or TelSeq. In fact, the only way to assess the accuracy of any telomere length assessment method should be based on measurements performed on a set of “artificial chromosomes” synthesized with telomeres, subtelomeric regions and interstitial telomeric repeats of known length. To our best knowledge, no experimental or computational method, including “gold standard” TRF has passed such validation. That is why the validity of Computel should be recognized in terms of correlations with other measures, and not the absolute values of mean telomere length estimates.

One of the important challenges in the assessment of telomere integrity is the determination of telomere lengths at individual chromosomes. Computel does not allow for that, since the telomeric pattern is not chromosome specific, and it is virtually impossible to identify the chromosome source of telomeric short-reads. Currently, there are no computational methods for individual telomere length assessment (including TelSeq), nor is it measured with TRF or qPCR experiments. There are few experimental techniques (qFISH [15], chemistry based methods [30]) that allow for obtaining telomere lengths from individual chromosomes. While the data derived from these experiments is important for genome stability assessment, mean telomere length has been proven to be informative as well, and associated with various biological phenomena, such as telomere position effect [31, 32], and disease association [16–19].

Even though Computel allows for overcoming the issues described above and has relatively high accuracy, it also has a number of limitations. Its most important limitation is an inability to handle variable telomeric patterns such as those characteristic to S. cerevisiae C_1-3_A/TG_1-3_ [33]. We intend to address this in future versions of Computel. A second limitation is that alignment, the most time-consuming step in the algorithm, is performed only with Bowtie 2. In the future, we will also consider implementation with other short-read alignment programs, such as BWA [34] and SOAP [35].

In conclusion, we have developed Computel, an open-source software package for estimating mean telomere length based on whole-genome NGS data. The overall results of the performance assessment demonstrate that this methodology is valid for mean telomere length association studies based on high-throughput data.

## Supporting Information

S1 Information Computel User Manual. The Computel User Manual, where detailed information about how to install and run Computel are provided.
(PDF)

S2 Information Additional results of Computel performance assessment. Assessment of Computel performance in various settings, and the mean telomere lengths assessed by Computel and TelSeq for the experimental data.
(DOC)

S3 Information Telseq recompilation details. The details describing how TelSeq was recompiled.
(DOC)

S4 Information Reads not captured by Computel, but passing TelSeq’s threshold of 7. (TXT)
