# Supplementary material for: Computel: Computation of Mean Telomere Length from Whole-Genome Next-Generation Sequencing Data
Source: PLoS One. 2015 Apr 29;10(4):e0125201. doi: 10.1371/journal.pone.0125201 (PMC4414351; doi:10.1371/journal.pone.0125201)
Supplement: S1 Information — The Computel User Manual, where detailed information about how to install and run Computel are provided. (PDF) [file pone.0125201.s001.pdf]

Computel User Manual

Introduction

Computel is R-based software for computation of mean telomere length from whole genome next generation sequencing (NGS) data.

Development

Computel has been developed by the members of the Bioinformatics Group at the Institute of Molecular Biology of the National Academy of Sciences of the Republic of Armenia (IMB NAS RA). You can visit the group's webpage at the following link: http://molbiol.sci.am/big.

Citation

When using Computel in your research, please refer to the GitHub repository at https://github.com/lilit-nersisyan/computel.

License

Copyright (C) 2014 Lilit Nersisyan & Arsen Arakelyan BIG IMB NAS RA.
This program is free software: you can redistribute and/or modify it under the terms of the GNU General Public License version 3. The license can be found at http://www.gnu.org/licenses/gpl.html.

Requirements

Computel is tested to work with 64 bit Windows 7, Linux and Mac OS X (10.6 and above).
The following software should be installed on your system:
R 3.0.3 or higher,
Bowtie2-2.1.0 (not 2.2.0!), http://sourceforge.net/projects/bowtie-bio/files/bowtie2/2.1.0/
Samtools 0.1.19, source: http://sourceforge.net/projects/samtools/files/samtools/0.1.19/ complied versions for Linux and Windows are included in the computel.zip file).
Picard tools 1.108 SamtoFastq.jar, http://sourceforge.net/projects/picard/ .

Computel has been tested only on provided versions of software. It does not work with Bowtie2-2.2.0, and is not guaranteed to work with higher versions of Samtools and Picard.

Download and installation

Computel can be downloaded from GitHub at https://github.com/lilit-nersisyan/computel or via the link to the full package.
Download and extract the scripts into a local directory. The folder contains examples with sample configuration files and scripts for execution of Computel either from command line ("computel.cmd.R") or from R environment ("computel.R"), and the main functional scripts ("pipeline.R", "functions.R", "validate.options.R").

Setting up samtools

Samtools source is available for download from http://www.htslib.org/. However, some of the samtools releases have a threshold of 8000 for maximum depth of coverage. This threshold should be increased for telomere length calculation, by adding the line "`bam_mplp_set_maxcnt(mplp,1000000);`" in the *bam2depth.c*, as indicated in the following patch:

```
Index: bam2depth.c
===================================================================
--- bam2depth.c     (revision 995)
+++ bam2depth.c     (working copy)
@@ -80,6 +80,7 @@

       // the core multi-pileup loop
       mplp = bam_mplp_init(n, read_bam, (void**)data); // initialization
+        bam_mplp_set_maxcnt(mplp,1000000); // set maxdepth to 1M
       n_plp = calloc(n, sizeof(int)); // n_plp[i] is the number of covering reads
from the i-th BAM
       plp = calloc(n, sizeof(void*)); // plp[i] points to the array of covering reads
(internal in mplp)
       while (bam_mplp_auto(mplp, &tid, &pos, n_plp, plp) > 0) { // come to the next
covered position
```

Precompiled Samtools binaries for Windows and Linux, as well as Picard SamToFastq.jar is also provided for convenience. Before running Computel, check whether the binary works on your system, and otherwise recompile it from source.

Runing Computel from R

Open "computel.R" and change the configuration variables in the script as appropriate (see *Configuration options* section). The *scripts.dir* should point to the directory where the "pipeline.R", "validate.options.R" and "functions.R" scripts are located. Finally, run the script.

Executing Computel from command line

Type the following in the command line:

```
Rscript path/to/computel.cmd.R path/to/config.file
```

In Windows, the path to Rscript.exe (usually, this is "C:\Program Files\R\R-3.x.x\bin") should be set as an environmental variable. Alternatively the full path to Rscript should be provided in the command line.

Algorithm description

The algorithm workflow is presented in the figure. For its detailed description, refer to the main paper (*citation*).

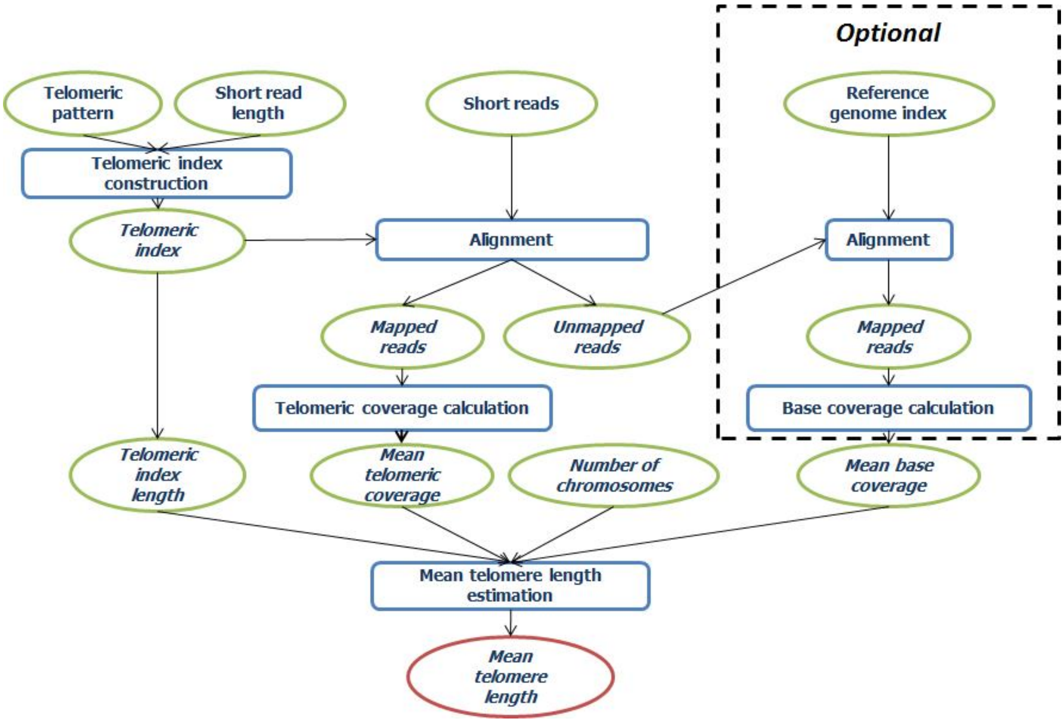

Configuration options

The following options should be specified before running Computel. These are given either in a configuration text file as an argument to "computel.cmd.R" script, or in the body of "computel.R" script in case of running Computel from R.

Value	Example	Description
**#directories**		
scripts.dir	D:/computel/	Path to directory containing "pipeline.R", "validate.options.R" and "functions.R" scripts.
bowtie.build.path	D:/bowtie2-2.1.0/bowtie2-build.exe	Path to bowtie2-build executable file or respective PATH variable, if applicable
bowtie.align.path	D:/bowtie2-2.1.0/bowtie2-align.exe	Path to bowtie2-align executable file or respective PATH variable, if applicable
samtools.path	D:/samtools-0.1.19/samtools.exe	Path to samtools executable file or respective PATH variable, if applicable
picard.samtofastq.jar	D:/picard-tools-1.108/SamToFastq.jar	Path to SamToFastq.jar or respective PATH variable, if applicable

**#input_reads**		
fastq1	tel_reads1.fq	Path to first pair FASTQ file in case of paired-end reads (option *single* should be set to *F*)
fastq2	tel_reads2.fq	Path to second pair FASTQ file in case of paired-end reads (option *single* should be set to *F*)
fastq	tel_reads.fq	Path to single FASTQ file in case of single-end reads (option *single* should be set to *T*)
single	F	*T*-single-end reads, *F* - paired-end reads.<br>Default is *T*.
read.length	76	Number of bases in short reads.<br>Default is *100*.
**#algorithm_options**		
pattern	TTAGGG	Sequence of single telomeric repeat.<br>Default is *TTAGGG*.
num.haploid.chr	23	Number of chromosomes in haploid genome.<br>Default is *23*.
min.seed	12	Minimum number of telomeric bases to be present in short reads for telomeric read alignment.<br>Default is *12*.
mode.local	F	Mode of alignment. *T* - local, *F* - end-to-end.<br>Default is *F.*
**#base_coverage_calculation_options**		
compute.base.cov	T	Specifies if the base coverage should be computed by read alignment to reference genome (*T*), or is supplied by the user (*F*).<br>Default is F.
base.cov	5.4	Precalculated mean coverage at reference genome. Given if option *compute.base.cov* is *F.*<br>Default is *1*.
base.index.pathtoprefix	base.index/base_index	Path to reference genome index directory and index

		prefix. Given if option *compute.base.cov* is *T.*
**#output_options**		
output.dir	output	Path to output directory.<br>Default is *./output*
**#system_options**		
num.proc	3	Number of processors to be used during alignment. Default is *3.*
**#additional_options**		
quals	--phred33	Quality scores of reads. Default is "--phred33". Alternatives are: –phred33, --phred64 or --solexa-quals. **Caution:** be careful when specifying quality score values (see the text).
ignore.err	F	Specifies whether error messages thrown by system calls to bowtie should be ignored. Default is *F.* **Caution:** if set to *T,* wrong results may be generated by computel, if there were errors in alignment or index building (see text).

Notes on some configuration options

Single vs. paired-end reads

Generally, Computel performs equally well with paired-end and single-end reads. Therefore, it is also possible to fuse the two read files into one single FASTQ file and set the **single** option to *T*.

Read length

Caution: If the **read.length** option is omitted or set to a wrong value, Computel will not warn the user and the algorithm will run, however, a wrong value of telomere length estimate will be outputted.

Pattern

Telomeric repeat pattern is specified for the 3'-end of the chromosome. E.g. in case of human telomeres, the sequence at 3'-end it is TTAGGG, while the 5'-end has pattern CCCTAA.
If the pattern is omitted in the configuration file, the default value TTAGGG for human telomere repeats will be used. In case of a different study organism another pattern should be specified.

Number of haploid chromosomes

Telomere length is calculated with the formula:

(mean(*tel.cov/base.cov*))*(rl+pl-1) / (2*num.haploid.chr*)*,

where *tel.cov* is coverage at telomeric index and *base.cov* is coverage at reference genome; *rl* is read length and *pl* is telomeric pattern length, *num.haploid.chr* is the number of haploid chromosomes.

The division by *num.haploid.chr* is for deriving at a mean value of telomere length for each chromosome end. The number 2 is to account for the two ends of each chromosome. Note that the ploidy of the genome will have no influence on the results, meaning that, adjusted for the rest of the parameters, the results will not differ for haploid, diploid and polyploid genomes.

Min.seed

This is an advanced option and specifies the minimum number of telomeric repeat bases. More specifically, this value is used for building the telomeric index with trailing N bases of length (*read.length* - *min.seed*), which is the “index.nontelomeric” region of the index in the figure below.

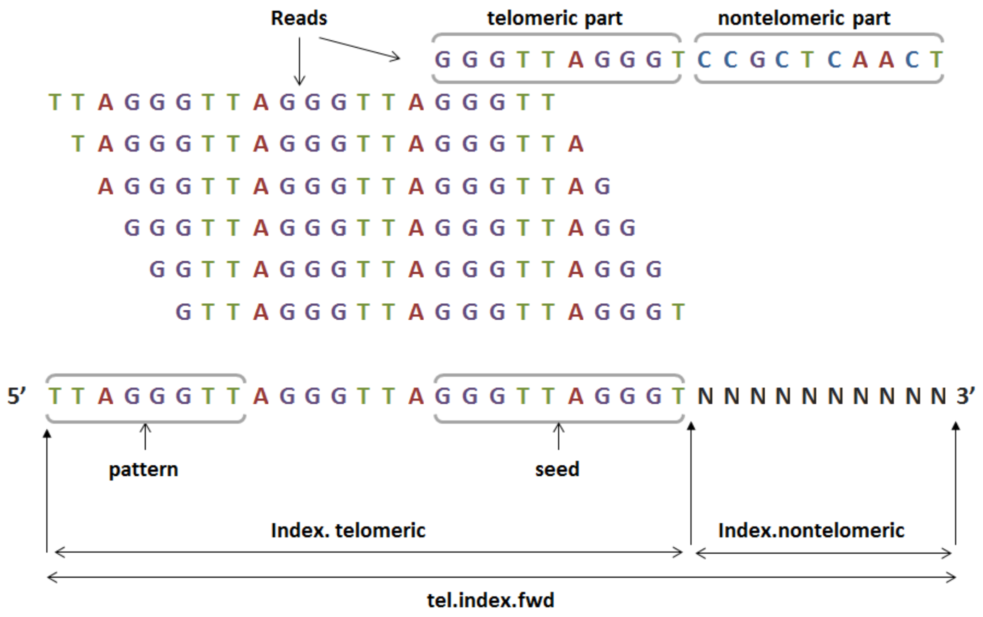

If this option is omitted, the default value of 12 will be used. Increasing **min.seed** will increase specificity but decrease sensitivity of capturing telomeric reads, and vice-versa.

We believe that for the majority of biological cases, the default value is optimal.

Alignment mode

When setting the **mode.local** value to T, the alignment of reads will be perfromed in Bowtie --local mode, instead of the default --end-to-end mode. Theoretically, local mode increases sensitivity of capturing telomeric reads. To our experience, for the majority of biological cases, the default --end-to-end mode is more accurate than the --local mode.

Base coverage calculation options

Alignment of the reads to reference genome is the most time consuming part of the algorithm. To our experience, the accuracy of the algorithm does not decrease if the base coverage is assessed beforehand with the formula

```
base.cov = (total number of reads)*(read length)/(total genome length)
```

and supplied to Computel with **base.cov** option. In that case, the **compute.base.cov** option should be set to *F*.
If the user wants the algorithm to compute the base coverage with alignment, the user should provide the prebuilt reference genome index via the **base.index.pathtoprefix** option and set the **compute.base.cov** option to *F*.

quals

The quality scores can differ in various experimental settings. By default, computel will assume bowtie's default option for quality scores. Alternatively, the user may choose the quality scores to either '--phred33', '--phred64' or '--solexa-quals'. **Caution:** if **ignore.err** option is set to *T*, and the specified quality score values are wrong, bowtie may fail to run the alignment by throwing a warning message, but telomere computation will be performed with incomplete alignment and wrong results will be generated. Thus, the user should be careful when specifying quality score values.

ignore.err

If this option is set to *F,* computel will halt further execution, if it receives error messages while performing system calls to bowtie align or build. Setting this option to *T,* will make computel ignore the error messages and continue telomere length calculation, which may result in wrong result generation. Therefore, the user should pay attention to the error messages thrown to command line and R consoles, after getting the results of the calculation.

Output format

The output is stored in the provided or default output directory. The telomeric index files are kept in "index" folder, the telomeric read alignment files are in "align" folder, and if base alignment files are kept in "base" folder, if generated. The final output is the "tel.length.xls" file, with the input parameters and estimated mean telomere length (*tel.length,* bp).

FAQ

The FAQ section will be generated once a question arises.
Feel free to ask questions and make suggestions by mailing to Lilit Nersisyan at l_nersisyan@mb.sci.am.
