# Supplementary material for: Computel: Computation of Mean Telomere Length from Whole-Genome Next-Generation Sequencing Data
Source: PLoS One. 2015 Apr 29;10(4):e0125201. doi: 10.1371/journal.pone.0125201 (PMC4414351; doi:10.1371/journal.pone.0125201)
Supplement: S2 Information — Assessment of Computel performance in various settings, and the mean telomere lengths assessed by Computel and TelSeq for the experimental data. (DOC) [file pone.0125201.s002.doc]

**S2 Supporting Information. Additional results of Computel performance assessment.**

## The influence of the Bowtie 2 alignment parameters on telomere length estimation

We have chosen the most appropriate short-read alignment parameters that would combine the maximal accuracy and speed of the alignment to the telomeric index by evaluating different alignment options offered by Bowtie 2 and comparing their performance.

The alignments were performed with the Bowtie 2 preset options for *--end-to-end* alignment, i.e. *--fast* (F), *--very-fast* (VF), *--sensitive* (S) and *--very-sensitive* (VS) modes. The argument for "mismatches in a seed alignment" (*–N*), was tested for both the default value of 0 and for the value of 1, since setting *-N* to 1 increases the alignment sensitivity. Next, the *-L* option, which is the length of the seed substrings to align during multi-seed alignment, was set to one third of read length, but with minimum value of 6 and maximum value of 22 (this means that if one third of read length is less than 6, it is assigned the value of 6, and if it is more than 22, it is set to 22). Finally, the *--n-ceil* option, which is the maximum number of allowed mismatches and ‘N’ bases in the alignment, was set to [*rl*-*min.seed*]*,* where *rl* is the short-read length and *min.seed* is the minimum number of telomeric read bases in the mapped reads. Thus, we ended up with 8 alignment modes (F-N0, VF-N0, S-N0, VS-N0, F-N1, VF-N1, S-N1, VS-N1) and compared their effect on the accuracy of telomere length estimation. Eventually, the mean relative error (MRE), standard error (SE) of MRE, root mean squared error (RMSE), and coefficient of determination (R^2^) were calculated, separately for single and paired-end reads, and compared across alignment modes.

The results showed that mean telomere length estimation was more accurate for those alignment modes, for which mismatches were allowed in the seed (F-N1, VF-N1, S-N1, VS-N1). For VS-N1, the MRE ± SE was -0.5 ± 0.17 % for single-end reads and -4.3 ± 0.01% for paired-end reads, RMSE was equal to 7% and 11%, respectively (Figure S1). Since the difference in performance among these four modes was very small and we were interested in higher sensitivity, we have chosen the VS-N1 as the default alignment option for Computel.


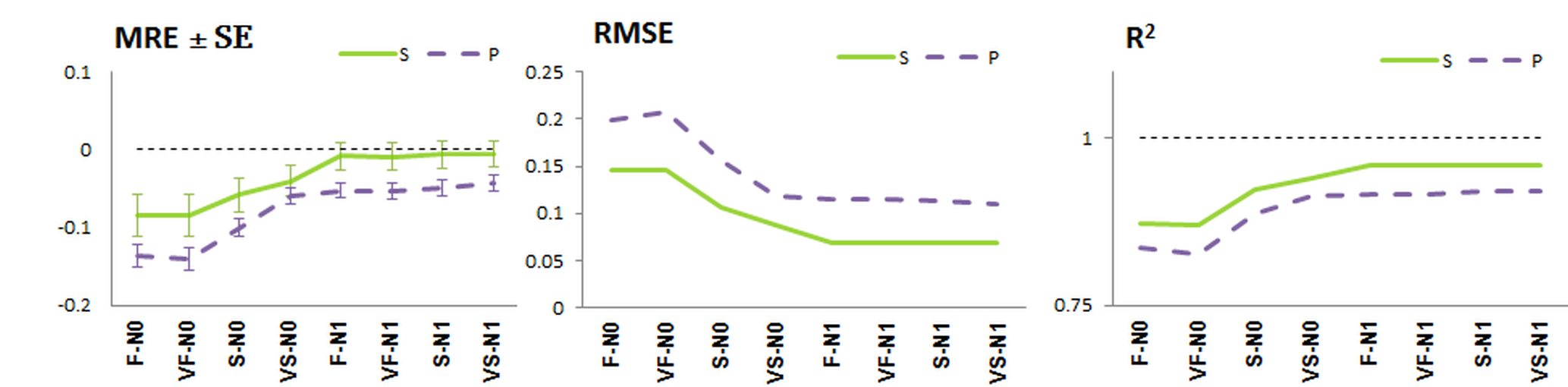


**Figure S1. Telomere length estimation accuracy in different alignment modes.**

S - single-end reads, P - paired-end reads.

Estimation of telomere length was performed with reads generated from 200 kb length region of human chromosome 1, with telomeres attached to both its ends with lengths sampled from a normal distribution with mean and SD equal to 10 kb and 7 kb . The modes of alignment (F-N0, VF-N0, S-N0, VS-N0, F-N1, VF-N1, S-N1, VS-N1), as well as read length, insert size and fold coverage ranges are described above. MRE – mean relative error between estimated and actual mean telomere lengths, RMSE – root mean squared error (error variance), R^2^ – coefficient of determination (quality of linear fit between estimated and actual mean telomere lengths).

## Performance assessment

### Comparison of performance for single-end reads

We have performed assessment of mean telomere length estimation accuracy depending on single-end read length (*rl*) and fold coverage (*fcov*), as described in *Methods, Algorithm validation with synthetic data* section of the manuscript.

According to multi-factor ANOVA test results, there were no significant differences in mean telomere lengths depending on read length, coverage or their combinations (Table S1).

**Table S1.** Effects of read length and coverage on MRE of mean telomere length estimation for single-end reads.

| **Variable** | **Type III Sum of Squares** | **df** | **F** | **Sig.** |
| --- | --- | --- | --- | --- |
| read length (*rl*) | 0.07 | 5 | 1.82 | 0.11 |
| fold coverage (fcov) | 0.02 | 6 | 0.48 | 0.83 |
| *rl-fcov* interaction | 0.21 | 30 | 0.95 | 0.54 |
| Error | 60.79 | 8358.00 | - | - |
| Total | 61.41 | 8400.00 | - | - |

From 42 *rl-fcov* combinations, in 21 cases the relative error of length estimate was not significantly different from 0, and in the rest, the calculated telomere length was underestimated by 0.2% - 0.3 % (Figure S2). Meanwhile, standard errors of MRE decreased along with the increase of fold coverage. Additionally, we observed decrease of relative error variance (RMSE), as well as improvement of linear fit (R^2^) with the increase in fold coverage, which is more pronounced for longer reads (Figure S2).


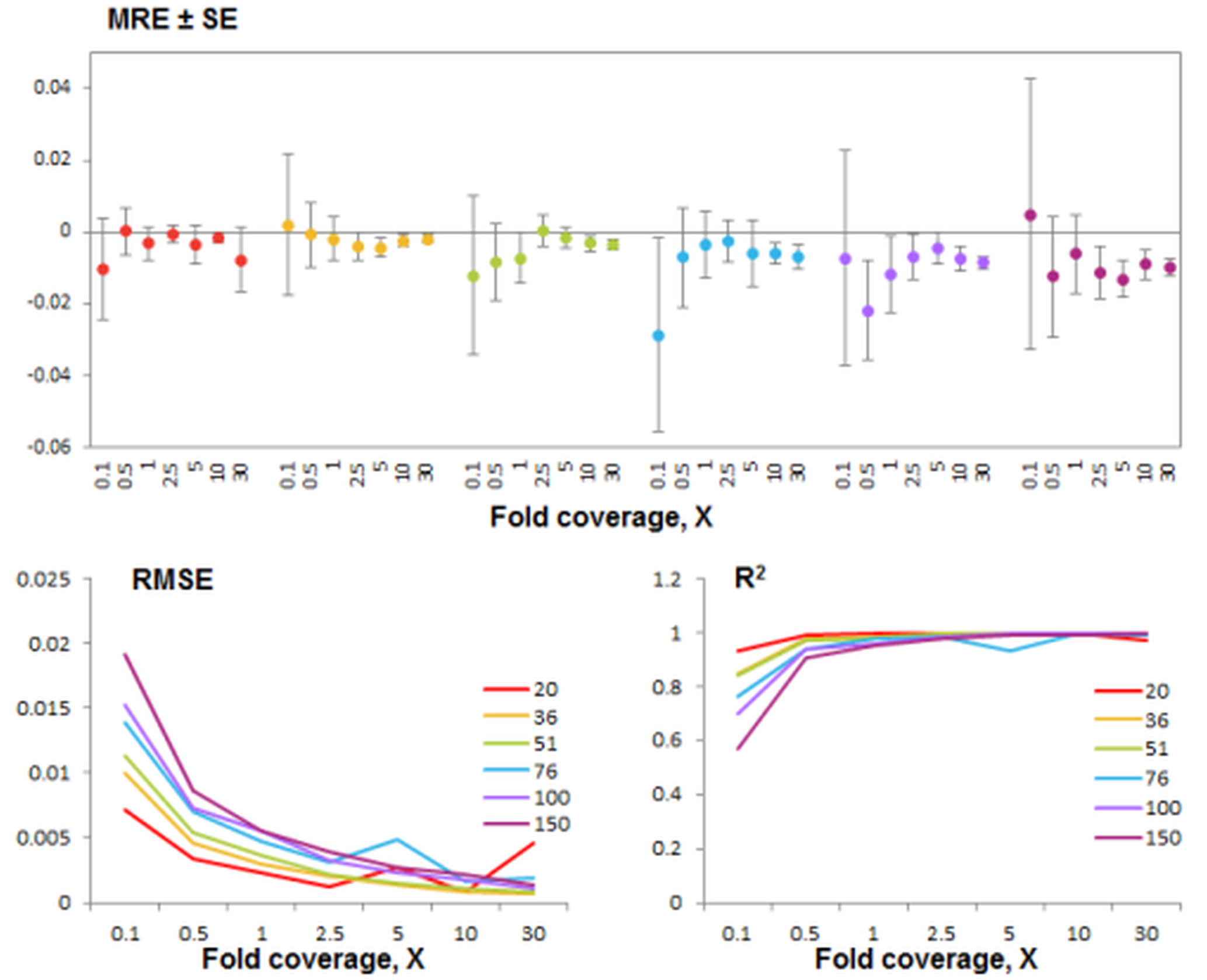


**Figure S2. Performance metrics of mean telomere length estimation for single-end reads depending on read length and coverage.**

Estimation of mean telomere length was performed with single-end reads generated from 200 kb length region of human chromosome 1, with telomeres attached to both its ends with lengths sampled from a normal distribution with mean equal to 10 kb and SD equal to 7 kb. Each read length is represented with one color. Dots on the first chart represent mean relative error (MRE) between estimated and actual mean telomere lengths; whiskers represent standard error (SE) of MRE. RMSE – root mean squared error (error variance), R^2^ – coefficient of determination (quality of linear fit between estimated and actual mean telomere lengths). Coverage is indicated on the x axes.

### Comparison of performance for paired-end reads

We have performed similar series of analyses for paired-end short-reads, accounting also for insert size effects, with scenarios described in *Methods, Validation scenarios* section of the manuscript.

Multi-factor ANOVA indicates that read length, coverage and insert size, as well as their pairwise interactions, do significantly influence relative error (MRE) of telomere length estimation in the case of paired-end reads (Table S2).

**Table S2. Effects of read length and coverage on MRE for paired-end reads.**

| **Variable** | **Type III Sum of Squares** | **df** | **F** | **Sig.** |
| --- | --- | --- | --- | --- |
| read length (*rl*) | 1.23 | 5 | 20.13 | 0.00 |
| fold coverage (*fcov*) | 0.18 | 6 | 2.43 | 0.02 |
| insert size (*mflen*) | 1.24 | 2 | 50.65 | 0.00 |
| *rl-fcov* interaction | 0.61 | 30 | 1.67 | 0.01 |
| *rl-mflen* interaction | 0.15 | 7 | 1.75 | 0.09 |
| *fcov-mflen* interaction | 0.46 | 12 | 3.10 | 0.00 |
| *rl-fcov-mflen* interaction | 0.53 | 42 | 1.03 | 0.43 |
| Error | 255.88 | 20895.00 | - | - |
| Total | 278.60 | 21000.00 | - | - |

From the 105 combinations of read length (rl), fold coverage (fcov) and insert size (mflen), in 12 cases the relative error of length estimate was not significantly different from 0, the calculated telomere length was significantly overestimated in two cases (by 2% and 5.5%), and underestimated in the rest (by 0.3% - 5.8 %) (Figure S3). Similar to the case of single end reads, mean relative error (MRE) decreased and quality of linear fit (R^2^) improved along with increas of fold coverage (Figure S4 and S5). For paired-end reads, telomere length estimation accuracy decreased with the increase in insert size (Figure S3).


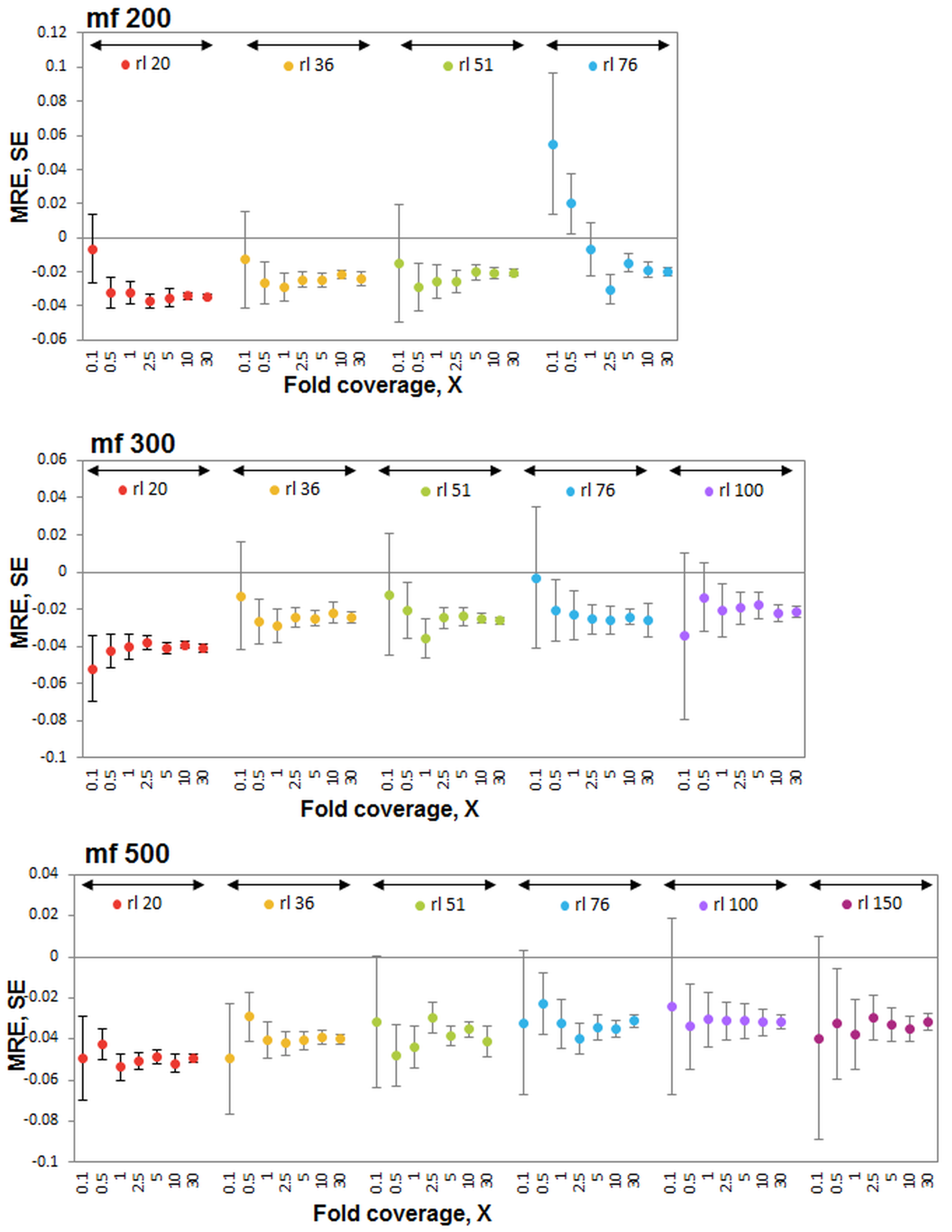


**Figure S3. Performance metrics of mean telomere length estimation for paired-end reads based on read length, coverage and insert size.**

Estimation of telomere length was performed with paired-end reads generated from 200 kb length region of human chromosome 1, with telomeres attached to both its ends with lengths sampled from a normal distribution with mean equal to 10 kb and SD equal to 7 kb. Insert sizes (200 nt, 300 nt and 500 nt) are indicated as chart titles. Each read length is represented with one color. Dots on the chart represent mean relative error (MRE) between estimated and actual mean telomere lengths; whiskers represent standard error (SE) of MRE. Coverage is indicated on the x axis.


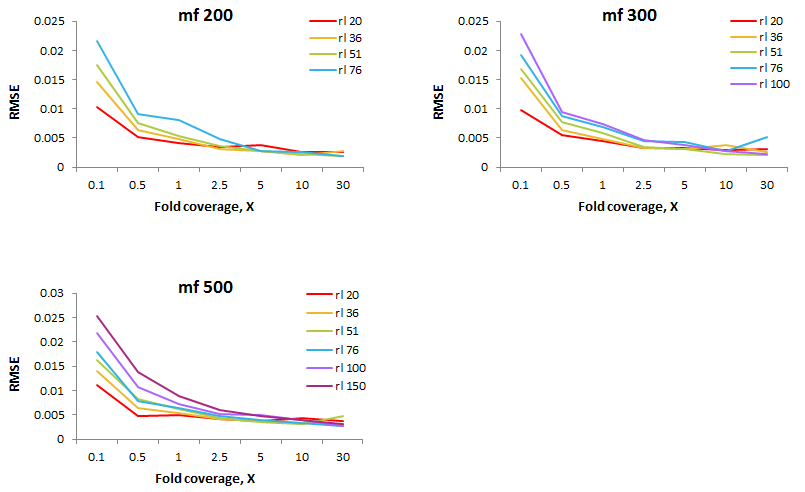


**Figure S4. Root mean squared error (RMSE) of telomere length estimation for paired-end reads based on read length, coverage and insert size.**

Estimation of telomere length was performed with paired-end reads generated from 200 kb length region of human chromosome 1, with telomeres attached to both its ends with lengths sampled from a normal distribution with mean equal to 10 kb and SD equal to 7 kb. Insert sizes (200 nt, 300 nt and 500 nt) are indicated as chart titles. Each read length is represented with one color. Coverage is indicated on the x axis.


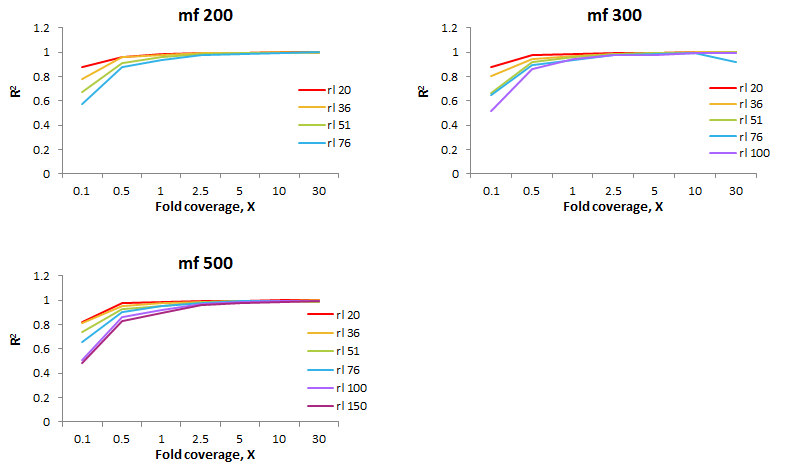


**Figure S5. Quality of linear fit (R^2^) of telomere length estimation for paired-end reads based on read length, coverage and insert size.**

Estimation of telomere length was performed with paired-end reads generated from 200 kb length region of human chromosome 1, with telomeres attached to both its ends with lengths sampled from a normal distribution with mean equal to 10 kb and SD equal to 7 kb. Insert sizes (200 nt, 300 nt and 500 nt) are indicated as chart titles. Each read length is represented with one color. Coverage is indicated on the x axis.

## Absolute values of mean telomere length estimates for neuroblastoma samples

**Table S3.** Mean telomere length (MTL) estimates by Computel and TelSeq for neuroblastoma samples

|  | Computel | | | TelSeq | | |
| --- | --- | --- | --- | --- | --- | --- |
|  | Cancer tissue (D), MTL, kb | Healthy tissue (N), MTL, kb | log_2_(D/N) | Cancer tissue (D), MTL, kb | Healthy tissue (N), MTL, kb | log_2_(D/N) |
| SJNBL001 | 24.0 | 4.4 | 2.45 | 28.9 | 6.0 | 2.27 |
| SJNBL002 | 13.8 | 4.5 | 1.61 | 18.7 | 6.1 | 1.61 |
| SJNBL009 | 3.2 | 7.0 | -1.12 | 4.4 | 9.9 | -1.16 |
| SJNBL030 | 2.8 | 5.4 | -0.95 | 4.0 | 7.9 | -1.00 |
| SJNBL031 | 7.6 | 3.6 | 1.08 | 10.8 | 4.9 | 1.12 |
