# Supplementary material for: Computel: Computation of Mean Telomere Length from Whole-Genome Next-Generation Sequencing Data
Source: PLoS One. 2015 Apr 29;10(4):e0125201. doi: 10.1371/journal.pone.0125201 (PMC4414351; doi:10.1371/journal.pone.0125201)
Supplement: S3 Information — The details describing how TelSeq was recompiled. (DOC) [file pone.0125201.s003.doc]

### S3 Supporting Information. Telseq recompilation details.

### Genome GC content length and chromosome number setup in TelSeq.

TelSeq performance and telomere length estimation accuracy relies on two constants, i.e. genome length at telomeric GC content (48-52%) and number of chromosomes in a haploid genome. These constants are hard-coded in TelSeq source (telseq.h and telseq.cpp) (Figure S1).


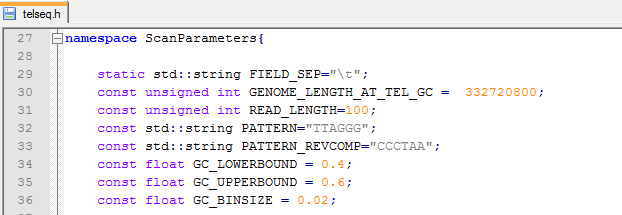


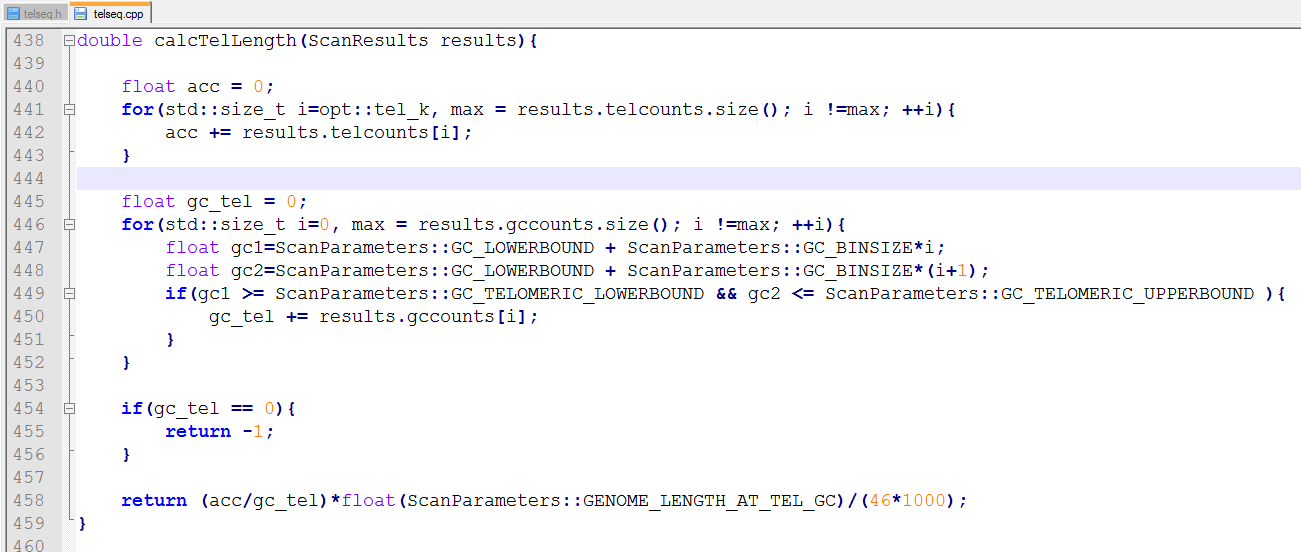


**Figure S3.1.** Hard coded constants for genome length with telomeric GC composition in telseq.h (above) and two chromosome ends times the number of chromosomes in a haploid genome (23) in telseq.cpp (below).

In order to compute telomere length for single human chromosome 1, we have computed this length of chromosome 1 regions with GC composition equal to 48-52%, using 1000 nt window, and derived a value of 21,722,000 for chromosome 1, instead of 332,720,800 for the total genome. Then we recompiled TelSeq with this new value for GENOME_LENGTH_AT_TEL_GC and 2 instead of 46 for the number of chromosome ends.
